# Proteome Profiling of Paulownia Seedlings Infected with Phytoplasma

**DOI:** 10.3389/fpls.2017.00342

**Published:** 2017-03-10

**Authors:** Xibing Cao, Guoqiang Fan, Yanpeng Dong, Zhenli Zhao, Minjie Deng, Zhe Wang, Wenshan Liu

**Affiliations:** ^1^Institute of Paulownia, Henan Agricultural UniversityZhengzhou, China; ^2^College of Forestry, Henan Agricultural UniversityZhengzhou, China

**Keywords:** *Paulownia tomentosa*, witches' broom, phytoplasma, transcriptome, iTRAQ, LC-MS/MS

## Abstract

Phytoplasma is an insect-transmitted pathogen that causes witches' broom disease in many plants. Paulownia witches' broom is one of the most destructive diseases threatening Paulownia production. The molecular mechanisms associated with this disease have been investigated by transcriptome sequencing, but changes in protein abundance have not been investigated with isobaric tags for relative and absolute quantitation. Previous results have shown that methyl methane sulfonate (MMS) can help Paulownia seedlings recover from the symptoms of witches' broom and reinstate a healthy morphology. In this study, a transcriptomic-assisted proteomic technique was used to analyze the protein changes in phytoplasma-infected *Paulownia tomentosa* seedlings, phytoplasma-infected seedlings treated with 20 and 60 mg·L^−1^ MMS, and healthy seedlings. A total of 2,051 proteins were obtained, 879 of which were found to be differentially abundant in pairwise comparisons between the sample groups. Among the differentially abundant proteins, 43 were related to Paulownia witches' broom disease and many of them were annotated to be involved in photosynthesis, expression of dwarf symptom, energy production, and cell signal pathways.

## Introduction

Paulownia, a fast-growing greening tree species, has many sought-after characteristics, including lightweight wood, high biomass production, and vigorous resprouting ability, which make them ideal environment protection trees. However, Paulownia is susceptible to a phytoplasma that causes Paulownia witches' broom (PaWB) disease, which gradually reduces biomass production (Doi et al., 1967). PaWB disease is usually characterized by stunted growth, witches' brooms, and phyllody, followed by dieback of branches and phloem necrosis (Namba, 2002).

Numerous studies have been carried out on Paulownia phytoplasma and the interaction of host with pathogen, and some advances at the physiological and chemical morphological, genetic, transcriptional, and epigenetic levels have been made (Doi et al., 1967; Fan and Jiang, 1997; Fan et al., 2007; Lin et al., 2009; Mou et al., 2013). The infecting phytoplasma lacks a cell wall and is bound only by a triple-layered unit membrane, which makes it difficult to culture *in vitro* (Doi et al., 1967). Although high-throughput sequencing and epigenetic technologies have been used to investigate the genes, microRNAs, and inherent metabolic pathways related to PaWB disease (Liu et al., 2013; Cao et al., 2014a,b; Fan et al., 2014, 2015a,b,c; Niu et al., 2016) however, understanding of the whole mechanisms of Paulownia—phytoplasma interactions is still lacking.

Proteins play important roles in catalyzing almost all chemical reactions, including those involved in plant growth and development, metabolism, and resisting pathogen invasion (Luge et al., 2014). Protein changes that occur in response to the interaction between Paulownia and phytoplasma have been studied using two-dimensional polyacrylamide gel electrophoresis (2D-PAGE; Fan et al., 2003); however, it is difficult to separate low abundance proteins and hydrophobic proteins using this technique (Molloy and Witzmann, 2002; Baggerman et al., 2005). Thus, 2D-PAGE cannot be used to characterize the profiles of all the proteins in cells or tissues; furthermore, the results are often difficult to reproduce. To help overcome some of the disadvantages of 2D-PAGE, several proteomic quantitative methods have been developed (Lodha et al., 2013). One such method is iTRAQ, which involves isobaric tagging of peptides for protein quantitation using mass spectrometry. iTRAQ can be used to identify and quantify hundreds of proteins in up to eight samples at one time with high sensitivity and accuracy (Fan et al., 2011; Wang B. et al., 2015), and has been successfully applied to detect changes in protein abundances in plants with pathogen infections (Taheri et al., 2011; Monavarfeshani et al., 2013; Dadakova et al., 2015).

Previously, we have shown that the symptoms of PaWB disease were reversed by treatment with a suitable concentration of methyl methane sulfonate (MMS) and that the phytoplasma was not detected in the recovered plants (Cao et al., 2014a; Fan et al., 2015a). In this study, we used iTRAQ to investigate protein changes in four groups of *Paulownia tomentosa* seedlings: healthy seedlings, phytoplasma-infected seedlings, and phytoplasma-infected seedlings treated with 20 or 60 mg·L^−1^ MMS. Proteins associated with PaWB disease were identified by searching against transcriptome sequences from the same samples. We identified a number of proteins that showed changes in abundance after infection by the PaWB phytoplasma, which may help in understanding the mechanisms associated with PaWB disease.

## Materials and methods

### Plant material and treatment

All samples in this study were tissue culture seedlings, healthy *P. tomentosa* seedlings (HP) and *P. tomentosa* seedlings infected with PaWB phytoplasma (PIP), both of them were obtained from the Institute of Paulownia. These seedlings were cultured for 30 days on 1/2 MS medium before being clipped (Murashige and Skoog, 1962). After clipping, the terminal buds of 1.5-cm PIP were transferred into 100-mL triangular flasks containing 20 ml 1/2 MS culture medium supplemented with 0, 20, or 60 mg·L^−1^ MMS (PIP, PIP-20, and PIP-60 respectively). The terminal buds of 1.5-cm HP were transferred into the same medium without MMS as the control. All the seedlings were cultured in a darkroom at 20°C for 5 days. After that, they were cultured at 25 ± 2°C under 130 μmol·m^−2^ s^−1^ light intensity with a 16/8 h light /dark photoperiod for 25 days. Then, the terminal buds of 1.5-cm seedlings in the four treatment groups were sheared, immediately frozen in liquid nitrogen, and stored at −80°C. For each treatment, 60 terminal buds were planted in 20 flasks. Each treatment was performed in triplicate.

### Extraction of protein from paulownia

The four samples (HP, PIP, PIP-20, and PIP-60) were ground to powder in liquid nitrogen, then these different powder were added the moderate lysis buffer with 1 mM PMSF and 2 mM EDTA, the details of the protein extraction method was described as Tang et al. (2016).

### iTRAQ labeling and strong cation exchange fractionation

To label the peptides obtained from the HP, PIP, PIP-20, and PIP-60 samples with iTRAQ reagent, 100 μg total protein from each sample solution was digested with Trypsin Gold (Promega, Madison, WI, USA). The peptides in the four samples were labeled with isobaric tags, the details method of iTRAQ-labeled according to the method of Meng et al. (2014). The labeled peptide mixtures were pooled and dried by vacuum centrifugation. Strong cation exchange (SCX) chromatography was performed with a LC-20AB HPLC Pump system (Shimadzu, Kyoto, Japan). The labeled peptide mixture contained proteins extracted from the PIP, PIP-20, PIP-60, and HP samples, including two technical replicates of isotopic labeling. Each biological replicate consisted of 12 terminal buds of the same stage from four different samples.

### LC-MS/MS analysis

The labeled peptide mixture was firstly resuspended and then the supernatant was loaded on a LC-20AD nano HPLC system (Shimadzu), then the data acquisition was performed with a TripleTOF 5600 System (AB sciex, concord, ON, Canada) fitted with a Nanospray III source (AB sciex) and a pulled quartz tip as the emitter (New Objectives, Woburn, MA). The mass spectrometer was operated with a RP of ≥30,000 full-width half-maximum for time-of-flight mass spectrometry scans. The method of the peptides were separated according to Qiao et al. (2012).

### Data analysis

Raw data files were firstly converted into Mascot generic format files using Proteome Discoverer 1.2 software (Thermo Fisher Scientific, Bremen, Germany), then these data were matched against the transcriptome database containing 105,812 all-unigene sequences (Fan et al., 2015a,b). For protein identification, to reduce the probability of false peptide identification, only peptides at the 95% confidence interval, as defined by Mascot probability analysis, greater than “identity” were considered as identified, and each confident protein involved at least one unique peptide. For protein quantitation, it was required that a protein contained at least two unique spectra.

To further identify the functions of the identified proteins, the Blast2GO program was run against the NCBI non-redundant protein sequence database to assign them Gene Ontology (GO) terms. The Kyoto Encyclopedia of Genes and Genomes (KEGG) and the Clusters of Orthologous Groups (COG) databases were used to classify and group the identified proteins. Quantitative protein ratios were weighted and normalized by the median ratio in Mascot. Proteins with a fold change ≥ 1.2 and *p* < 0.05 were determined as differentially abundant proteins.

### Correlation analysis of the transcriptome and proteome data

In this section, the correlation analysis of the transcriptome and proteome data was carried out. Firstly, the proteins identified by iTRAQ were matched to 105,812 unigene sequences of the transcriptome. After that, the proteins which have homologs in the transcritome were screened out. Then we calculated the number of the correlated protein in four different comparisons, including the PIP vs. PIP-20 comparison, PIP-20 vs. PIP-60 comparison, HP vs. PIP-60 comparison and HP vs. PIP comparison, and the correlated proteins can be considered as the identified proteins which have been expressed at the transcription level. Subsequently, the quantitative analysis of the correlated proteins was performed, and the criterion of the quantitation protein is the unique peptide ≥2. According to the results of the quantitative analysis, we further picked out the differentially expressed proteins (fold change ≥ 1.2 and *p* < 0.05) in different comparisons. At last, the number of the genes that corresponding to the correlated proteins and the number of differential expressed proteins were figured out. The other important correlation analysis is to compare the abundance levels of the differentially expressed proteins and their corresponding genes from the transcriptome sequencing.

### Comparison scheme of proteins associated with PaWB

A comparison scheme of PaWB-related proteins was described by Liu et al. (2013). The proteins were searched against the NCBI non-redundant protein sequence database using the Blast2GO program to assign GO terms, and against the COG and KEGG databases to classify them to pathways and functional groups. To deeply analyze the function of PaWB-related proteins, protein BLAST (https://blast.ncbi.nlm.nih.gov/Blast.cgi) was used to confirm the function of these proteins, then the functional classification of them was performed.

### Quantitative real-time RT-PCR analysis

RNA samples from HP, PIP, PIP-20, and PIP-60 were extracted with Trizol (Sangon, Shanghai, China). First-strand cDNAs of the RNA samples were synthesized using an iScript cDNA synthesis kit (Bio-Rad, Hercules, CA, USA). From the transcriptome data (Fan et al., 2015a,b), we randomly chosen 12 unigene sequences that corresponded to the differentially abundant proteins for qRT-PCR, primers for each of the unigenes were designed using PrimerPremier 5.0 software (PREMIER Biosoft International, Palo Alto, Calif). The results were analyzed using the 2^−ΔΔCt^ method (Livak and Schmittgen, 2000). The expression level of each unigene was analyzed in three replicates. All the primers sequences used for the qRT-PCR are listed in Supplementary Table S1. Statistical analysis was performed using SPASS 19.0 (SPASS, Inc., Chicago, IL, USA).

## Results

### Identification of basic protein information by iTRAQ analysis

**Figure 1:**
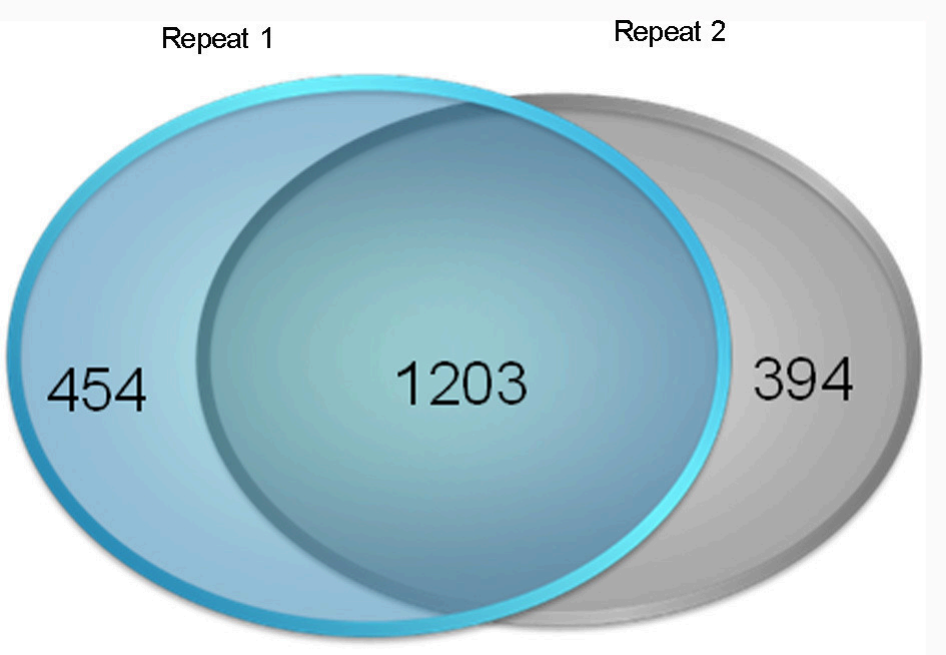
**Results of the reproducibility of protein identification**. Repeat 1: the first repetition of protein identification. Repeat 2: the second repetition of protein identification.

**Figure 2:**
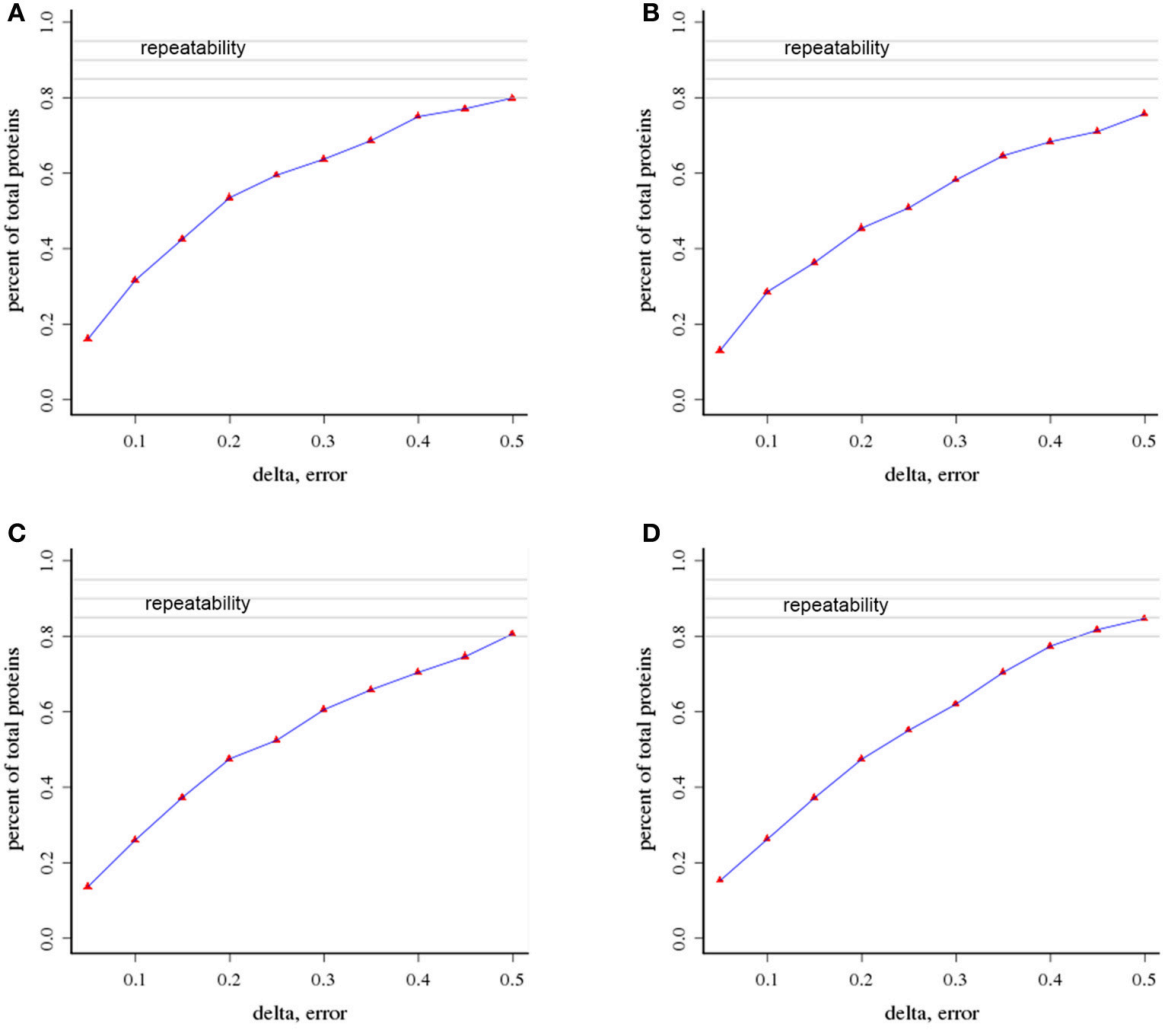
**Analysis of the repeatability of protein identification (A)** HP vs. PIP; **(B)** HP vs. PIP-60 **(C)** PIP-20 vs. PIP-60; **(D)** PIP vs. PIP-20.

We used an iTRAQ approach to analyze the total proteins in four *P. tomentosa* samples (HP, PIP, PIP-20, and PIP-60; Table 1). Protein homologs were identified against the transcriptome sequencing data from the same samples. We obtained 386,933 total spectra by combination analysis, and 21,948 of the spectra were matched using Mascot software. Among the 21,948 matched spectra, 17,811 were unique. The total number of detected proteins was 2,051 (Supplementary Table S2). The accuracy of the mass spectrometry was <2 ppm, and the database search peptides matching error was below 0.05 Da. We carried out two biological replicates to identify the total proteins and found that 1,597 of the proteins were identified in repeat 1, 1,657 were identified in repeat 2, and 1,203 were common to the two sets (Figure 1). The producibility of the proteomic analysis showed that the results for the duplicates were of high quality (Figure 2), which indicated that the proteome results were reliable.

**Table 1:** **Statistic of protein identification**.

**Group name**	**Total spectra**	**Spectra**	**Unique spectra**	**Peptide**	**Unique peptide**	**Protein**
Repeat 1	182,895	10,381	8,500	3,773	3,324	1,597
Repeat 2	204,038	11,567	9,311	3,986	3,460	1,657
Combination	386,933	21,948	17,811	—	—	2,051

*Repeat 1: the first repetitiion of protein identification. Repeat 2: the second repetition of protein identification*.

### Functional classification of the identified proteins

**Figure 3:**
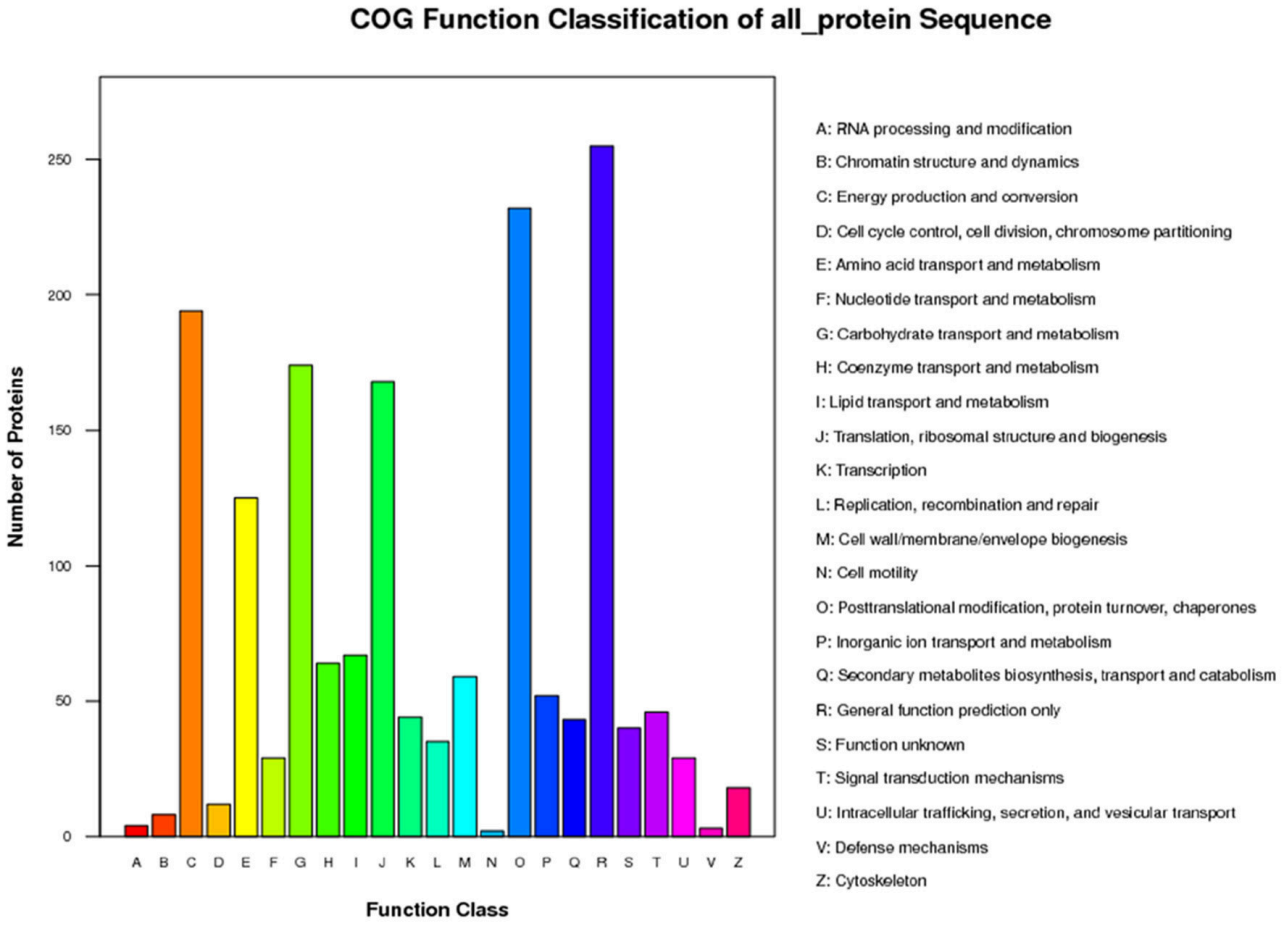
**COG classification of all protein**.

**Figure 4:**
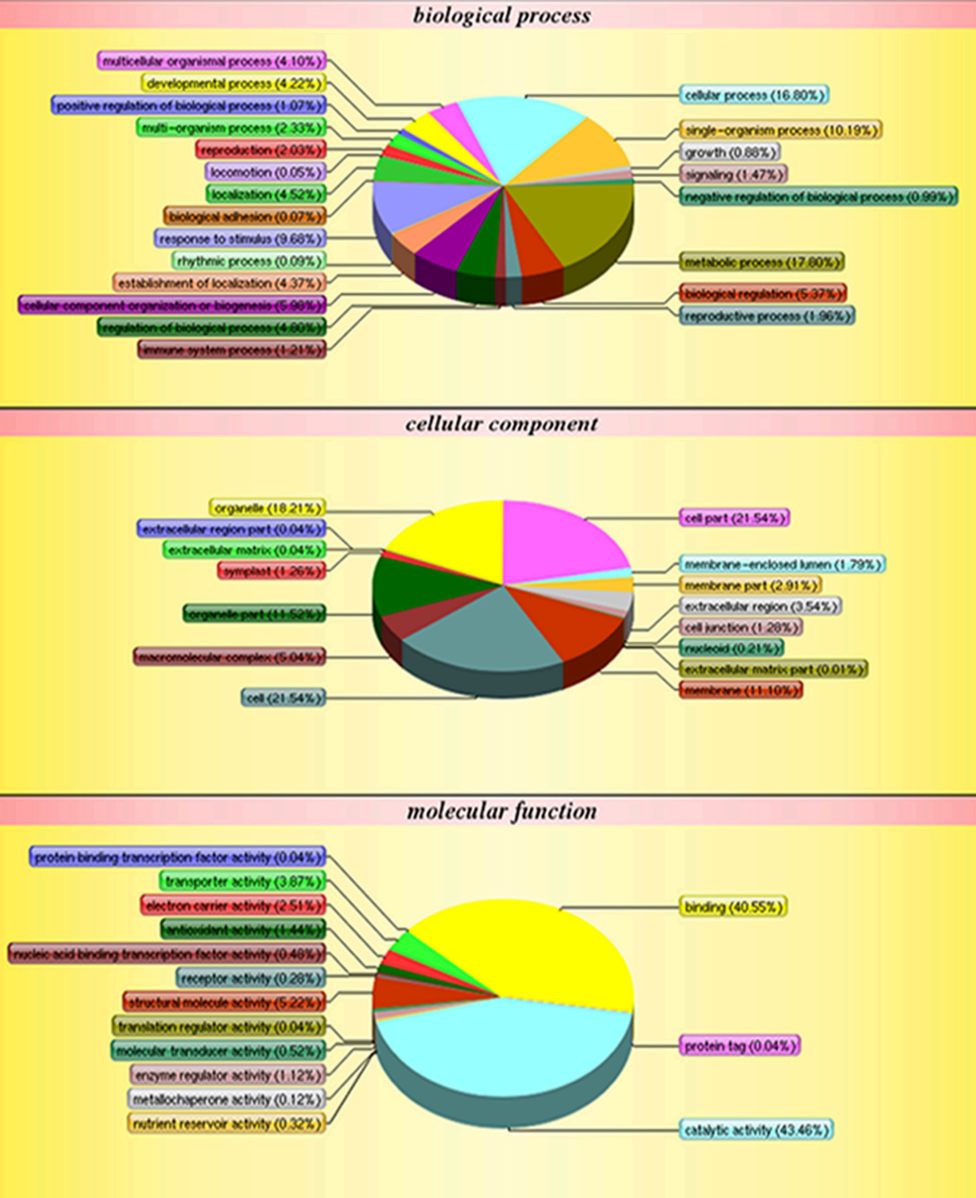
**GO classification of all protein**.

The functions of the 2,051 identified proteins were classified using COG. The proteins were classified into 23 COG subgroups (Figure 3), among which “general function prediction only,” “post-translational modification, protein turnover, chaperones,” and “energy production and conversion” were significantly enriched and contained the largest numbers of proteins, while “RNA processing and modification,” “defense mechanisms,” and “cell motility” contained the smallest numbers of proteins (Supplementary Table S3). The 2,051 proteins were also classified into the three GO categories: biological process, molecular function, and cellular component (Figure 4). Under biological process, “metabolic process” contained the largest number of proteins; under cellular component, “cell” and “cell part” contained the largest numbers of proteins; and under molecular function, “catalytic activity” contained the largest number of proteins (Supplementary Table S4). In the KEGG pathways analysis (Supplementary Table S5), the 2,051 proteins were mapped to 117 biological pathways of which “metabolic pathways,” “biosynthesis of secondary metabolites,” and “ribosome” were the most enriched metabolism pathways, while “linoleic acid metabolism,” “monoterpenoid biosynthesis,” and “circadian rhythm-plant” were the least enriched pathways. Based on these results, we concluded that most of the annotated proteins may affect metabolic processes, post-translational modification, protein turnover, chaperones, and catalytic activity.

### Analysis of differentially abundant proteins between the diseased and recovered seedlings

Differentially abundant proteins (DAPs) were defined as proteins having a 95% confidence level, *p* < 0.05, and fold changed >1.2. According to these criteria, the abundance of 1,172 of the 2,051 proteins did not differ significantly in the PIP vs. HP, PIP-60 vs. PIP-20, PIP-20 vs. PIP, and PIP-60 vs. HP comparisons, while the abundance of 879 of the proteins changed significantly (Table 2). Among the 879 DAPs, 223 were identified in PIP vs. HP, 204 were identified in PIP-60 vs. PIP-20, 278 were identified in PIP-20 vs. PIP, and 174 were identified in PIP-60 vs. HP. Further analysis indicated that most of the DAPs were correlated to several metabolism pathways, namely biosynthesis of secondary metabolites, photosynthesis, carbon fixation in photosynthetic organisms, carotenoid biosynthesis, nitrogen metabolism, porphyrin, and chlorophyll metabolism, glyoxylate and dicarboxylate metabolism, pyrimidine metabolism, porphyrin, and chlorophyll metabolism, and plant–pathogen interaction.

**Table 2:** **The number of differently abundance proteins**.

**Comparations**	**Numbers of up-regulated proteins**	**Numbers of down-regulated proteins**	**Numbers of differently abundance proteins**
PIP *vs*.PIP-20	121	157	278
PIP-20 *vs*.PIP-60	105	99	204
HP *vs*.PIP-60	85	89	174
HP *vs*.PIP	122	101	223

### Correlation of transcriptome and proteome data

A correlation analysis of the transcriptome and proteome data was carried out, and the numerical relationships between the correlated proteins and the total proteins were calculated in the different comparisons. In the PIP vs. PIP-20 comparison, 2,050 proteins were correlated with the transcriptional data; among them, 1,046 proteins were quantitated and 278 of them were differentially abundant. In the PIP-20 vs. PIP-60 comparison, 2,048 proteins were correlated with the transcriptional data; among them 1,051 proteins were quantitated and 204 of them were differentially abundant. In the HP vs. PIP-60 comparison, 2,049 proteins were correlated with the transcriptional data; among them, 1,083 proteins were quantitated and 174 of them were differentially abundant. In the HP vs. PIP comparison, 1,073 proteins were quantitated and 223 of them were differentially abundant. All the numbers of these proteins corresponding genes were shown in Table 3.

**Table 3:** **Correlation analysis of transcription and proteome**.

**Comparation**	**Type**	**Numbers of proteins**	**Numbers of genes**
PIP vs.PIP-20	The correlated proteins	2,050	104,021
	Quantitation	1,046	–
	Differential expression	278	5,461
PIP-20 vs.PIP-60	The correlated proteins	2,048	105,420
	Quantitation	1,051	–
	Differential expression	204	18,769
HP vs.PIP-60	The correlated proteins	2,049	105,217
	Quantitation	1,083	–
	Differential expression	174	21393
HP vs.PIP	The correlated proteins	2,051	102,661
	Quantitation	1,073	–
	Differential expression	223	2,821

Based on these results, we compared the abundance levels of the proteins and the expression levels of their corresponding genes. The following patterns were detected: (1) the same trend between the corresponding mRNA and protein levels; (2) the opposite trend between the corresponding mRNA and protein levels: (3) no change in protein levels, but changes in gene expression levels; (4) changes in protein levels, but no change in gene expression levels. These results show that the protein abundance level is not necessarily reflected in the expression level of the corresponding mRNA. Similar results have been reported in phytoplasma-infected Mexican lime (Monavarfeshani et al., 2013).

### Analysis of DAPs related to PaWB

According to the comparison scheme of PaWB-related proteins, a total of 43 DAPs were obtained that changed their abundance levels in response to phytoplasma infection (Table 4), then the function of 43 DAPs were further classified in the COG, GO, and KEGG databases. The DAPs were classified into 13 COG categories (Supplementary Table S6), among which the three largest groups were “energy production and conversion,” “post-translational modification, protein turnover, chaperones,” and “general function prediction only.” The 43 DAPs were assigned 38 GO terms (Supplementary Table S7); 20 were under biological process, 11 were under cellular component, and five were under molecular function. In the KEGG pathway enrichment analysis, the 43 DAPs were mapped to 27 pathways (Supplementary Table S8), among which the two largest groups were “metabolic pathways” and “biosynthesis of secondary metabolites,” while the two smallest groups were “plant-pathogen interaction” and “phenylpropanoid biosynthesis,” indicating that changes in some metabolites may be important in the response of *P. tomentosa* seedlings to PaWB infection.

**Table 4:** **List of identified and characterized PaWB related to proteins in other species**.

**Accession**	**HP vs. PIP**	**Annotation**	**Gene resources**	**Function classification**	**References**
CL51.Contig1_All	Up	20S proteasome beta subunit	Arabidopsis	Post-translational modification, protein turnover, chaperones	Dielen et al., 2011
CL2295.Contig5_All	Up	Ubiquitin extension protein	Jatropha	Post-translational modification, protein turnover, chaperones	Tao et al., 2015
CL6965.Contig2_All	Up	Subunit of chloroplasts chaperonins	Arabidopsis,	Post-translational modification, protein turnover, chaperones	Zhang et al., 2016
CL9610.Contig1_All	Up	Peptidyl-Prolyl Isomerase	Arabidopsis,	Post-translational modification, protein turnover, chaperones	Bissoli et al., 2012
CL1409.Contig1_All	Up	Protein grpE	Arabidopsis	Post-translational modification, protein turnover, chaperones	Hu et al., 2012
CL5562.Contig2_All	Up	fk506- and rapamycin-binding protein	Arabidopsis,	Post-translational modification, protein turnover, chaperones	Xiong and Sheen, 2012
CL9305.Contig3_All	Down	Component of the light harvesting complex	Arabidopsis	Photosynthesis	Bressan et al., 2016
Unigene11985_All	Down	Chloroplast thylakoid lumen protein	Arabidopsis	Photosynthesis	Wang et al., 2016; Zhu, 2016
CL13475.Contig1_All	Down	Photosystem I subunit E-2	Arabidopsis	Photosynthesis	Ihnatowicz et al., 2007
Unigene12214_All	Down	Alpha carbonic anhydrase	Arabidopsis	Photosynthesis	Zhurikova et al., 2016
CL13475.Contig2_All	Down	Subunit E of photosystem I	Arabidopsis	Photosynthesis	Ihnatowicz et al., 2007
CL8582.Contig1_All	Down	Plastid-specific ribosomal protein 4	Arabidopsis	Photosynthesis	Tiller et al., 2012
CL12525.Contig1_All	Up	Ca2+-binding protein	Arabidopsis	Cell signal transduction	Zhou et al., 2013
Unigene12188_All	Up	Ca2+-binding protein	Arabidopsis	Cell signal transduction	Zhou et al., 2013
CL473.Contig1_All	Up	ATP synthase gamma subunit	Arabidopsis	Energy metabolism	Budak et al., 2013
CL13354.Contig2_All	Down	NADP-malic enzyme	Arabidopsis	Energy metabolism	Tronconi et al., 2008
Unigene11563_All	Up	Subunit of glyceraldehyde-3-phosphate dehydrogenase	Arabidopsis	Energy metabolism	Guo et al., 2014
Unigene22259_All	Up	Pectin methylesterase	Arabidopsis	Plant defense	Bethke et al., 2014
CL13450.Contig1_All	Up	Aldo/keto reductase	Jatropha, rice	Plant defense	Mudalkar et al., 2016
Unigene29082_All	Up	Cinnamyl-alcohol dehydrogenase	Medicago	Plant defense	Zhao et al., 2013
CL3972.Contig3_All	Up	Fasciclin-like arabinogalactan protein	Populus,	Plant defense	Wang H. et al., 2015
CL7387.Contig1_All	Up	Germin-like protein	Arabidopsis	Plant defense	Beracochea et al., 2015
CL8516.Contig2_All	Up	Protein tyrosine phosphatases	Rice	Carbohydrate transport and metabolism	Singh et al., 2010
CL8015.Contig1_All	Up	Glycerophosphoryl diester phosphodiesterase-like protein	Arabidopsis	Glycerol metabolism	Hayashi et al., 2008
CL12982.Contig1_All	Up	Disulfide isomerase-like protein	Arabidopsis	Cell redox homeostasis	Wittenberg et al., 2014
CL9983.Contig2_All	Up	GDSL-motif esterase	Arabidopsis	Carboxylic ester hydrolase	Meyer et al., 2012
Unigene32404_All	Up	Plastid-lipid associated protein	Arabidopsis	Abscisic acid-mediated photoprotection	Youssef et al., 2010
CL399.Contig1_All	Up	Subunit of magnesium chelatase	Barley	Coenzyme transport and metabolism	Braumann et al., 2014
CL4384.Contig3_All	Up	Ubiquitin-fold modifier 1-like isoform	Arabidopsis	Phosphatidylinositol biosynthetic process	Paula and Williams, 2016
CL4374.Contig1_All	Up	Rubisco small subunit	Arabidopsis	Photorespiration	Ido et al., 2015
Unigene18956_All	Up	Rubisco small subunit	Arabidopsis	Photorespiration	Ido et al., 2015
CL3902.Contig3_All	Down	Plasma membrane H+-ATPase	Arabidopsis	Inorganic ion transport and metabolism	Yamauchi et al., 2016
CL13468.Contig1_All	Down	Lipid-transfer protein-like protein	Arabidopsis	Lipid transport	Edstam and Edqvist, 2014
CL11604.Contig1_All	Up	Acetyl-CoA carboxylase BCCP subunit	Arabidopsis	Fatty acid biosynthesis	Salie et al., 2016
Unigene764_All	Up	Glycine-rich RNA-binding protein	Arabidopsis	General function prediction	Yang et al., 2014
CL4020.Contig3_All	Up	A linker histone like protein	Saccharomyces	DNA binding	Georgieva et al., 2015
CL6642.Contig1_All	Up	Ribosomal protein L6 family protein	Escherichia coli	Translation, ribosomal structure and biogenesis	Shigeno et al., 2016
Unigene4318_All	Up	Histone H2A	Arabidopsis	Chromatin structure and dynamics	Yelagandula et al., 2014
CL6457.Contig2_All	Up	Enhancer of sos3-1	Tobacco	rRNA processing	Li et al., 2013
Unigene10439_All	Up	Membrane-associated progesterone binding protein 2	Arabidopsis	Steroid binding	Yang et al., 2005
Unigene21155_All	Up	Unknown/hypothetical	———–	Unknown/hypothetical	———–
CL5467.Contig1_All	Up	Unknown/hypothetical	———–	Unknown/hypothetical	———–
CL13502.Contig1_All	Up	Unknown/hypothetical	———–	Unknown/hypothetical	————

To confirm and dig out the function of the 43 DAPs, protein sequence BLAST (https://blast.ncbi.nlm.nih.gov/Blast.cgi) was used to confirm the functional of the 43 DAPs in other plant. The result showed 40 DAPs functions have been confirmed in other plants (Table 4). Of these proteins, six proteins were classified in the section named as post-translational modification, protein turnover, chaperones, such as, 20S proteasome beta subunit, ubiquitin extension protein, subunit of chloroplasts chaperonin, peptidyl-Prolyl Isomerase, Co-chaperone GrpE family protein and fk506- and rapamycin-binding protein; six proteins participated in photosynthesis pathway, including chloroplast thylakoid lumen protein (TLP), photosystem I subunit E-2 (PSI-E2), plastid-specific ribosomal protein 4 (*PSRP4*), the component of the light harvesting complex (LHCI) and alpha carbonic anhydrase (CA), and the subunit E of photosystem I (PSI-E2); three proteins were associated with energy metabolism pathway, such as ATP synthase gamma subunit, NADP-malic enzyme (NADP-ME), and the subunit of glyceraldehyde-3-phosphate dehydrogenase (GAPC); two Ca^2+^-binding protein involved in cell signal transduction pathways; five proteins, included aldo/keto reductase, cinnamyl-alcohol dehydrogenase, fasciclin-like arabinogalactan protein, germin-like protein, and pectin methylesterase took part in plant defense. In addition these DAPs with functions, 3 of PaWB-related proteins were annotated as unknown function, their function still need to verify.

### qRT-PCR analysis

**Figure 5:**
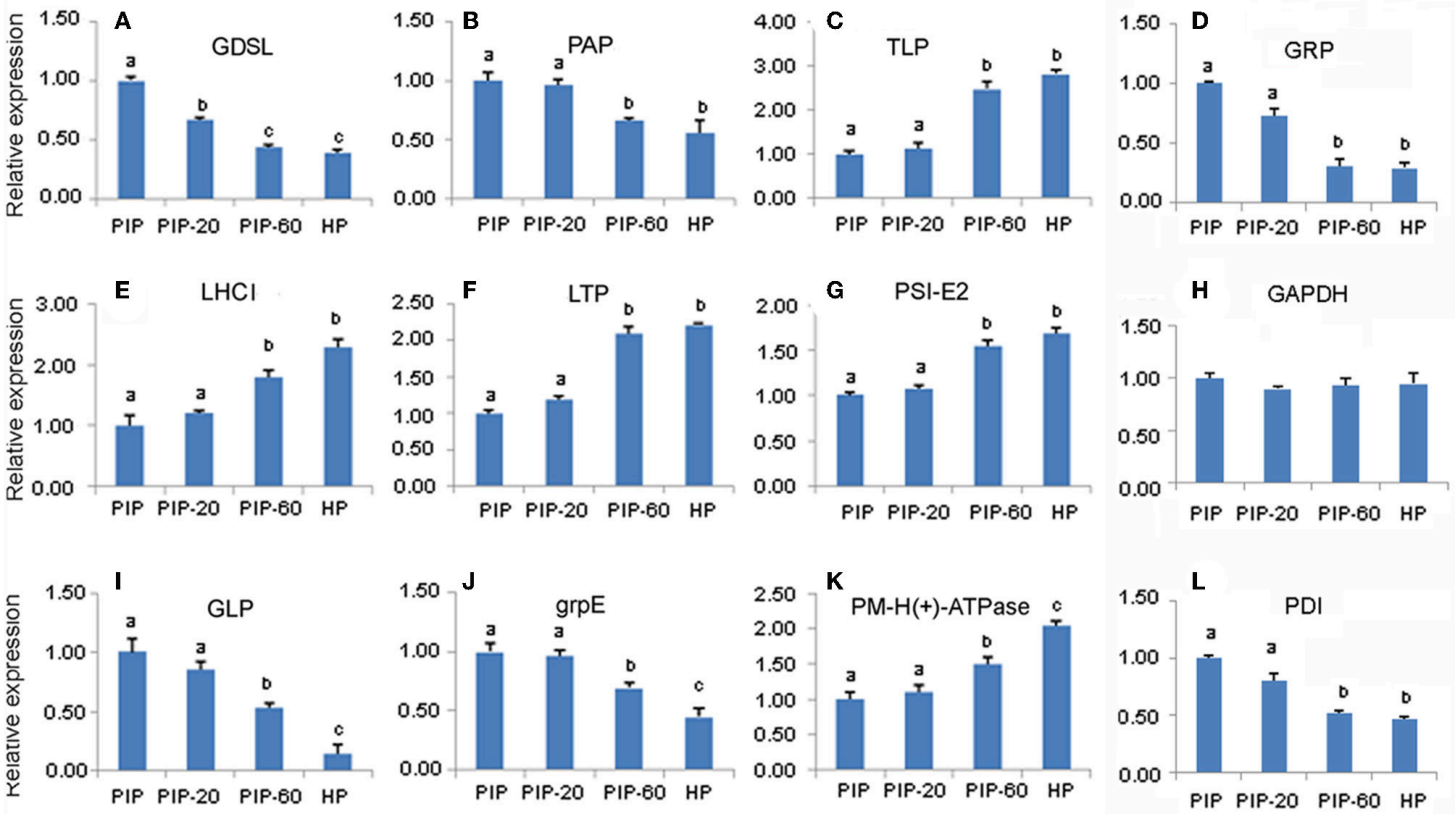
**Quantitative RT-PCR analysis of ***P. tomentosa*** candidate gene. (A)** mRNA levels for GDSL; **(B)** mRNA levels for PAP; **(C)** mRNA levels for TLP; **(D)** mRNA levels for GADPH; **(E)** mRNA levels for LHCI; **(F)** mRNA levels for LTP; **(G)** mRNA levels for PSI-E2; (**H)** mRNA levels for GRP; **(I)** mRNA levels for GLP; (**J)** mRNA levels for grp E; **(K)** mRNA levels for PM-H(+)-ATPase; **(L)** mRNA levels for PDI.

To confirm the results of the transcriptome sequencing data, 12 gene sequences corresponding to PaWB-related proteins were selected randomly for the qRT-PCR assays (Figure 5). The results showed that the relative expressions of six of the 12 DAP-related unigenes were significantly down-regulated in the process of the morphometric recovery, while five were significantly up-regulated. These 11 DAP-related expression patterns were consistent with the results of the iTRAQ LC-MS/MS analysis. For one of DAPs, no related mRNA expression was detected. This discrepancy may be attributed to post-transcriptional processing, post-translational processing and modification, or to different rates of degradation of mRNA and protein.

## Discussion

Information about the molecular basis of the Paulownia response to phytoplasma infection is still meager. For many years, our group has investigated the molecular mechanisms of PaWB and reported changes at the physiology, biochemistry, morphology, and molecular levels in the host Paulownia plants (Fan et al., 2003, 2014, 2015a,b,c; Liu et al., 2013; Cao et al., 2014a,b; Steinhorst and Kudla, 2014; Niu et al., 2016). We identified some PaWB-related genes and functionally classified them, detected changes in DNA methylation levels and patterns, and discovered some important proteins related to PaWB disease. Even so, many relevant aspects of this disease still need to be addressed. In this study, we adopted a transcriptomic-assisted proteomic approach to investigate protein changes associated with the interaction of *P. tomentosa* and phytoplasma. We obtained a total of 2,051 proteins by searches against the transcriptome data (Fan et al., 2015a,b). According to our previous scheme, the correlation analysis between the transcriptome and proteome data identified 43 DAPs potentially related to PaWB. These DAPs were predicted to be involved in photosynthesis, energy metabolism, lipid metabolism, the calvin cycle, glycolysis/ gluconeogenesis, epigenetic modification, plant resistance, and signal transduction.

### Phytoplasma infection decrease the expression of major components of photosynthesis

The onset of the yellowish leaves symptom in phytoplasma-infected plants is one of the most obvious symptoms of witches' broom. This symptom is closely related to changes in photosynthesis, and many studies have reported that photosynthesis is arrested in phytoplasma-infected plants, as evidenced by a decrease in pigment molecules such as chlorophyll b levels soon after infection (Scarpari et al., 2005). Accordingly, phytoplasma infection leads to a reduction in the expression of genes that encode photosystem components, further suppressing photosynthesis (Ji et al., 2009; Margaria et al., 2012; Nejat et al., 2015). Similar results have been reported in the interaction of Paulownia and phytoplasma, where several important protein components associated with photosynthesis were found to be significantly differentially abundant. These components include two proteins in photosystem I, six proteins in photosystem II, two proteins in light harvesting complex I, pyruvate kinase, and the pI6.8 24-kDa protein (Fan et al., 2003; Mou et al., 2013; Liu et al., 2013).

In this study, we identified six DAPs participated in photosynthesis, including *TLP*, two PSI-E2s, *PSRP4*, LHCI, and CA, which had reduced abundance levels in response to phytoplasma infection.

In the phytoplasma-infected seedlings, we detected one DAP, *PSRP 4* was down regulated, which belongs to photosystem II. Tiller et al. (2012) reported that *Arabidopsis* RNAi *PSRP 4* mutant appeared light-green phenotype, smaller mesophyll cells, and the chlorophyll content, chloroplast translation, plastocyanin content, and maximum quantum efficiency of photosystem II (F_V_/F_M_) significantly reduced, and the mutants also showed strongly reduced accumulation of three complexes (photosystem II, cytochrome b6f complex, photosystem I), which were responsible for photosynthetic electron transport.

There were three DAPs belong to Photosystem I (PSI), including TLP, LHCI, and PSI-E2. It is well known that thylakoid lumens in both integral and soluble membrane proteins, and TLP take part in light harvesting and electron transfer in the photosynthetic chain. In this study, the TLP was down-regulated in the diseased seedlings. Evidences showed that the reduction of TLP not only damaged the thylakoid electron flow, but also decreased chlorophyll a content and further slacked the photosynthesis (Wang et al., 2016; Zhu, 2016), this result is consistent with the previous RNA-seq results, which showed that a reduction in photosynthesis of phytoplasma-infected plants could be attributed to the loss of several thylakoid membrane proteins (Mou et al., 2013). At the same time, the expression of LHCI was reduced, which severely affected the light reactions in Photosystem I (PSI) (Bressan et al., 2016). Interestingly, we also detected a decreased abundance of PSI-E2. Ihnatowicz et al. (2007) demonstrated that the rate of thylakoid electron transfer were not affected in the absence of PSI-E2. Therefore, we speculate that the decreased abundance of the thylakoid lumenal protein in the PaWB-infected *P. tomentosa* plants might partly explain the yellowish leaves symptom.

Another DAP associated with photosynthesis was CA, which had reduced abundance level in the phytoplasma-infected seedlings. This protein plays a vital role in the early event of photosynthesis. Zhurikova et al. (2016) indicated that the reduction of the activity of CA affected the effective quantum yield of photosystem II, thus, its low abundance might weaken photosynthesis in the phytoplasma-infected plants.

### Phytoplasma infection induced the expression of proteins related to dwarf symptom

The hormonal imbalance in plants is the most factors for the abnormal symptom. Hoshi et al. (2009) discovered that TENGU can interfere the expression of auxin-related gene, which was the main reason for the plant proliferation and dwarf symptom. In the interaction of paulownia and phytoplasma, Mou et al. (2013) indicated that the key enzymes of cytokinin biosynthesis including isopentenyl diphosphate isomerase and isopentenyltransferase were significantly induced in the phytoplasma-infected Paulownia; Liu et al. (2013) demonstrated that the auxin efflux carrier 5NG4 were down-regulated in the phytoplasma-infected seedlings, which resulted in auxin accumulation in the paulownia.

In our proteome analysis, the abundance of two proteins that related to gibberellin (GA) and brassinosteroid (BR) were changed after phytoplasma infection: for example, the abundance of the glycine rich protein *GRP* was increased in phytoplasma-infected seedlings. It has been shown that over-abundance of *GRP* reduced the activity of several intermediates of the GA biosynthetic pathway, and transgenic *Arabidopsis* (*At*GRP) displayed reduced length of the vegetative stem (Löhr et al., 2014). The abundance of the membrane steroid binding protein (MSBP) also increased after phytoplasma infection. MSBP encodes a 220-amino acid protein that can bind to 24-epi-brassinolide and negatively regulates BR signaling, and overexpression of *MSBP* could inhibit cell elongation through downregulating cell elongation related genes, and result in the reduced cell elongation and shortened hypocotyl (Yang et al., 2005). Based on the results above, we concluded that the differentially abundant *GRP* and *MSBP* affected GA and BR biosynthesis, which were linked to the short internodes and dwarf morphology.

### Phytoplasma infection disturb the energy metabolism balance of the host

Phytoplasma genomes lack many metabolic pathways, making them unlikely to synthesize nucleotides, and they lack the key gene for ATP synthesis, they may need to import ATP from their host (Carle et al., 2011). Therefore, in phytoplasma-infected plants, the host must supply enough energy for intense phytoplasma growth. In phytoplasma-infected mulberry, the host's energy production was decreased (Ji et al., 2009). Oshima et al. (2001) demonstrated that, in onion, phytoplasma strongly depended on the host's glycolysis pathway to obtain more energy, while, in phytoplasma-infected Mexican lime, energy production by the TCA cycle was significantly enhanced (Monavarfeshani et al., 2013).

In this study, we observed that the abundance levels of some key proteins related to energy metabolism in the phytoplasma-infected *P. tomentosa*, such as ATP synthase gamma subunit, NADP-ME, and GAPC. In the phytoplasma-infected *P. tomentosa* plants, the ATP synthase γ subunit showed increased abundance. The ATP synthase γ subunit is closely related to the ATP production, and the up-regulation of ATP synthase γ subunit can enhance the photosynthetic rates in stressed plant (Budak et al., 2013). It is well known that the activity of chloroplast CF0-CF1-ATP synthase can be regulated by the light-dark. Kohzuma found that the modified γ subunit by mutating conserved D211V, E212L, and 226L acidic amino acids only altered the light induced regulation, but not metabolism induced regulation, and verified the function mutants of γ subunits affected photosynthetic ATP synthesis (Kohzuma et al., 2012, 2013). Therefore, in the photosynthesis pathway, the infected paulownia ATP might exhibit a tendency of increasing.

The other pathway of energy production is TCA cycle, an important pathway of energy production, in our study, the expression of DAP like NADP-ME showed decreased abundance, which plays a central role in the metabolite flux through the TCA cycle. In the Arabidopsis *nadp-me* knockout mutant, the contents of the TCA metabolites like 2-oxoglutarate and succinate were increased, while that of citrate and fumarate were decreased (Tronconi et al., 2008), implying that differential expression of NADP-ME had little impact on TCA cycle, and energy production by TCA cycle was not the necessary pathway for phytoplasma propagation in the host. This result was in disagreement with that in phytoplasma-infected Mexican lime (Monavarfeshani et al., 2013), the difference may be depend on the type of phytoplasmas and their hosts.

Another pathway of energy production is glycolytic pathway. GAPC, an important cytosolic enzyme which catalyzes a key reaction in glycolysis, was up-regulated in the phytoplasma infected seedlings. Evidence showed that GAPC play important roles in energy and carbohydrate metabolites, and Guo et al. (2014) reported that knockout or overexpression of GAPCs caused significantly changes in the content of intermediates of glycolytic pathway, the ratios of ATP/ADP, and NAD(P)H/NAD(P).

Compared with the DAPs functions of the three energy production pathways above, we supposed that glycolytic and photosynthesis may be the main energy provider for Paulownia response to the phytoplasma. However, details of the actual process need to be further researched.

### Phytoplasma infection evokes complicated cell signal transduction pathways in the host

The involvement of cell signal transduction pathways in plant—pathogen interactions directly or indirectly affected plant development (Ranjan et al., 2015). In Paulownia—phytoplasma interactions, many cell signal-related proteins have been identified from the transcriptome level, including plant hormones, calcium-dependent protein kinases, MAP kinases, receptor-like kinases, LRR receptor-like serine/threonine-protein kinases, L-ascorbate peroxidase, (S)-2-hydroxy-acid oxidase, and nitricoxide synthase (Mou et al., 2013; Liu et al., 2013; Cao et al., 2014a,b; Fan et al., 2015b). In our proteome analysis, we detected several DAPs mainly involved in Ca^2+^, ROS and plant defense signal pathway.

Calcium, an ubiquitous secondary messenger, plays an important role in all aspects of cell function, which has been regarded as versatile intracellular signal (Steinhorst and Kudla, 2014). Calcium-binding proteins is the component of the calcium-signaling pathway. In this study, two Ca^2+^-binding proteins were increased after phytoplasma infection. Zhou et al. (2013) reported that pathogen can manipulate the host Ca^2+^ signaling machinery to benefit their own life cycles. At the same time, evidence also has been documented that the calcium-binding protein could be as an effector taking part in plant defense (Ye et al., 2017), demonstrating that calcium-signaling pathway plays a central role in the interaction of paulownia and phytoplasma.

The abundance of the germin-like protein GLP, which belongs to a large ubiquitous family of plant glycoproteins, was also increased in the PaWB-infected seedlings. GLP plays a vital role in plant defense (Rietz et al., 2012). Further, it has been reported that high levels of GLP may initiate oxidative bursts in pathogen-infected plants and elevate the levels of endogenous reactive oxygen species (ROS) (Beracochea et al., 2015). Increasing evidence has shown that ROS (and redox signals) not only can induce cell damage, but also can act as reactive substrates to kill pathogens (Nejat et al., 2015). However, the ROS production was regulated by antioxidant protein disulfide isomerase (PDI), which was induced after phytoplasma infection. PDI, a major ER protein, usually acts as a molecular chaperone and component of signal-transduction pathways. It has been reported that PDI can limit potential cell damage by ROS generation after pathogen infection in plants (Stolf et al., 2011), which has been implicated in the complex interplay of defense-related signaling pathways.

Keeping the above views in mind, our results indicate that the response of *P. tomentosa* to PaWB infection involved several interconnected signaling pathways, including Ca^2+^ and ROS-mediated signaling, and plant defense signaling, which coordinate the plant's response to phytoplasma. Regulation of cell signal pathways need different proteins that are induced by interactions with susceptible as well as resistant hosts, which play positive or negative roles in the Paulownia response to phytoplasma, depending on the speed and intensity of the interaction responses.

## Conclusions

In this study, we combined transcription and proteome analyses to investigate changes in protein abundances in *P. tomentosa* plants in response to phytoplasma infection. The results revealed complex interactions between the Paulownia plants and the phytoplasma, which will contribute substantially to our understanding of the still largely unknown mechanisms that underlie the pathogenicity of phytoplasma. By analyzing the data, we obtained 2,051 proteins, 879 of which were differentially abundant, and 43 of them were found to be related to PaWB. Most of these proteins were predicted to participate in photosynthesis, energy production, and cell signal pathways. Based on the functional analysis of DAPs, we concluded that PaWB infection might lead to decreased photosynthesis, induced the expression of proteins related to dwarf symptom, unbalanced host energy metabolism, as well as abnormalities in cell signal transduction. Together, our data contribute to better understanding the mechanisms associated with PaWB. Future challenges will be to validate the roles of individual proteins and explore their functions in the regulation of the Paulownia response to phytoplasma.

## Ethics statement

This article does not contain any studies with human participants or animals performed by any of the authors.

## Availability of supporting data

All sequencing data generated in this study is available from the SRA-Archive (http://www.ncbi.nlm.nih.gov/sra) under the study accession SRP057771 and SRP068599. The 4 cDNA libraries SRA accession number are as follows: SRS924899 (HP), SRS924915 (PIP), SRS1252326 (PIP-20) and SRS924916 (PIP-60).

## Author contributions

GF conceived and designed the experiments. XC wrote the paper. YD and WL analyzed the data. ZZ performed the experiments. MD and ZW contributed reagents and analysis tools.

## Funding

This work was supported by the fund of the Transformation Project of the National Agricultural Scientific and Technological Achievement of China (2012GB2D000271), the Central Financial Forestry Science Promotion Project (GTH [2012]01), the Fund of the Technology Innovation Team Project of Zhengzhou (121PCXTD515) and the Fund of Zhongyuan Scholarship Foundation of Henan Province (122101110700).

### Conflict of interest statement

The authors declare that the research was conducted in the absence of any commercial or financial relationships that could be construed as a potential conflict of interest.
