# Supplementary material for: Proteome Profiling of Paulownia Seedlings Infected with Phytoplasma
Source: Front Plant Sci. 2017 Mar 10;8:342. doi: 10.3389/fpls.2017.00342 (PMC5344924; doi:10.3389/fpls.2017.00342)
Supplement: Supplementary file 1 [file Table1.DOCX]

| Gene name | Function of the proteins | Forward primer sequence (5‘ - 3’) | Reverse primer sequence (5‘ - 3’) |
| --- | --- | --- | --- |
| 18S |  | ACATAGTAAGGATTGACAGA | TAACGGAATTAACCAGACA |
| Pt.CL9983 | GDSL-motif esterase (GDSL) | CAGGAACAGGACCACTTG | TGTATTAGCAGCAATGAAGAC |
| Pt.Unigene32404 | Plastid-lipid-associated protein (PAP) | CGCTCCTCCTACTCCTTC | ACTCATCCTCGTCCTTCG |
| Pt.Unigene11985 | chloroplast thylakoid lumen protein (TLP) | AGAGAAGACAGAGGTTGATG | GTAAGAGTTCACAGGCAATG |
| Pt.CL9305 | component of the light harvesting complex ( LHCI ) | GGCTATGCTTGCTGTTCC | GCTCCTCTGGTGCTCTAC |
| Pt.CL13468 | lipid-transfer protein-like protein (LTP) | ACCTGCTAATATCTCCGACTG | CCTCCATCACTCCTCACATC |
| Pt.Unigene11563 | subunit of glyceraldehyde-3-phosphate dehydrogenase (GAPDH) | GCTGCTTCATTCAACATCATTC | AGGTCAACTACAGAAACATCAAC |
| Pt.CL13475 | photosystem I subunit E-2 ( PSI-E2) | ATTGGATGAAGTGGAAGTGGTTG | GGTGTTGAAGTGAATGAATGTAGC |
| Pt.CL12982 | Protein disulfide isomerase (PDI) | AGATTGTTATTGTTGGAGTGTTTC | GTTGGCTTGCTGACTGATTC |
| Pt.Unigene764 | Glycine-rich RNA-binding protein (GRP) | CAAGAGTTATTACTGACAGAGAC | CCACCAGCATTGTTAAAGC |
| Pt.CL7387 | Germin-like protein (GLP) | GTCATTCACTCTACTTATCTTCAC | ATCACTCACCGTTACATTAGC |
| Pt.CL1409 | Protein grpE(grpE) | AGCAGAGATGGAGAATGTGAAG | AGCAGAAGAAGCCCTACCC |
| Pt.CL3902 | Plasma membrane H+-ATPase (PM-(H+) -ATPase) | TGGACTTCTGCTGGTGACTG | CGGATGGCGAACTTCATTAGG |

**Table S-1 Primers used for *P. tomentosa* DEPs corresponding gene qRT-PCR analysis**
