# Supplementary material for: Proteome Profiling of Paulownia Seedlings Infected with Phytoplasma
Source: Front Plant Sci. 2017 Mar 10;8:342. doi: 10.3389/fpls.2017.00342 (PMC5344924; doi:10.3389/fpls.2017.00342)
Supplement: Supplementary file 3 [file Table3.DOCX]

| Code | Functional categories | Number of all protein |
| --- | --- | --- |
| A | RNA processing and modification | 4 |
| B | Chromatin structure and dynamics | 8 |
| C | Energy production and conversion | 194 |
| D | Cell cycle control, cell division, chromosome partitioning | 12 |
| E | Amino acid transport and metabolism | 125 |
| F | Nucleotide transport and metabolism | 29 |
| G | Carbohydrate transport and metabolism | 174 |
| H | Coenzyme transport and metabolism | 64 |
| I | Lipid transport and metabolism | 67 |
| J | Translation, ribosomal structure and biogenesis | 168 |
| K | Transcription | 44 |
| L | Replication, recombination and repair | 35 |
| M | Cell wall/membrane/envelope biogenesis | 59 |
| N | Cell motility | 2 |
| O | Posttranslational modification, protein turnover, chaperones | 232 |
| P | Inorganic ion transport and metabolism | 52 |
| Q | Secondary metabolites biosynthesis, transport and catabolism | 43 |
| R | General function prediction only | 255 |
| S | Function unknown | 40 |
| T | Signal transduction mechanisms | 46 |
| U | Intracellular trafficking, secretion, and vesicular transport | 29 |
| V | Defense mechanisms | 3 |
| Z | Cytoskeleton | 18 |

Table S3 COG functional classification of all protein
