# Supplementary material for: Proteome Profiling of Paulownia Seedlings Infected with Phytoplasma
Source: Front Plant Sci. 2017 Mar 10;8:342. doi: 10.3389/fpls.2017.00342 (PMC5344924; doi:10.3389/fpls.2017.00342)
Supplement: Supplementary file 4 [file Table4.DOCX]

Table S4 GO function classification of all protein

| Ontology | Classification | Number of all protein |
| --- | --- | --- |
| biological_process | biological adhesion | 6 |
|  | biological regulation | 434 |
|  | cellular component organization or biogenesis | 483 |
|  | cellular process | 1357 |
|  | developmental process | 341 |
|  | establishment of localization | 353 |
|  | growth | 71 |
|  | immune system process | 98 |
|  | localization | 365 |
|  | locomotion | 4 |
|  | metabolic process | 1437 |
|  | multi-organism process | 188 |
|  | multicellular organismal process | 331 |
|  | negative regulation of biological process | 80 |
|  | positive regulation of biological process | 86 |
|  | regulation of biological process | 388 |
|  | reproduction | 164 |
|  | reproductive process | 158 |
|  | response to stimulus | 782 |
|  | rhythmic process | 7 |
|  | signaling | 119 |
|  | single-organism process | 823 |
| cellular_component | cell | 1638 |
|  | cell junction | 97 |
|  | cell part | 1638 |
|  | extracellular matrix | 3 |
|  | extracellular matrix part | 1 |
|  | extracellular region | 269 |
|  | extracellular region part | 3 |
|  | macromolecular complex | 383 |
|  | membrane | 844 |
|  | membrane part | 221 |
|  | membrane-enclosed lumen | 136 |
|  | nucleoid | 16 |
|  | organelle | 1385 |
|  | organelle part | 876 |
|  | symplast | 96 |
| molecular_function | antioxidant activity | 36 |
|  | binding | 1017 |
|  | catalytic activity | 1090 |
|  | electron carrier activity | 63 |
|  | enzyme regulator activity | 28 |
|  | metallochaperone activity | 3 |
|  | molecular transducer activity | 13 |
|  | nucleic acid binding transcription factor activity | 12 |
|  | nutrient reservoir activity | 8 |
|  | protein binding transcription factor activity | 1 |
|  | protein tag | 1 |
|  | receptor activity | 7 |
|  | structural molecule activity | 131 |
|  | translation regulator activity | 1 |
|  | transporter activity | 97 |
