# Supplementary material for: Proteome Profiling of Paulownia Seedlings Infected with Phytoplasma
Source: Front Plant Sci. 2017 Mar 10;8:342. doi: 10.3389/fpls.2017.00342 (PMC5344924; doi:10.3389/fpls.2017.00342)
Supplement: Supplementary file 6 [file Table6.DOCX]

| Code | Functional categories | Number of the protein related to PaWB |
| --- | --- | --- |
| A | Chromatin structure and dynamics | 1 |
| B | Energy production and conversion | 8 |
| C | Cell cycle control, cell division, chromosome partitioning | 2 |
| D | Carbohydrate transport and metabolism | 4 |
| E | Coenzyme transport and metabolism | 1 |
| F | Lipid transport and metabolism | 1 |
| J | Translation, ribosomal structure and biogenesis | 1 |
| H | Cell wall/membrane/envelope biogenesis | 2 |
| I | Posttranslational modification, protein turnover, chaperones | 7 |
| J | Inorganic ion transport and metabolism | 2 |
| K | General function prediction only | 5 |
| L | Signal transduction mechanisms | 2 |
| M | Cytoskeleton | 2 |

Table S6 COG functional classification of the protein related to PaWB
