# Supplementary material for: Proteome Profiling of Paulownia Seedlings Infected with Phytoplasma
Source: Front Plant Sci. 2017 Mar 10;8:342. doi: 10.3389/fpls.2017.00342 (PMC5344924; doi:10.3389/fpls.2017.00342)
Supplement: Supplementary file 7 [file Table7.DOCX]

Table S7 GO function classification of the protein related to PaWB

| Ontology | Classification | Number of the protein related to PaWB |
| --- | --- | --- |
| biological_process | biological adhesion | 2 |
|  | biological regulation | 13 |
|  | cellular component organization or biogenesis | 16 |
|  | cellular process | 31 |
|  | developmental process | 8 |
|  | establishment of localization | 5 |
|  | growth | 2 |
|  | immune system process | 4 |
|  | localization | 5 |
|  | metabolic process | 32 |
|  | multi-organism process | 5 |
|  | multicellular organismal process | 8 |
|  | negative regulation of biological process | 1 |
|  | positive regulation of biological process | 2 |
|  | regulation of biological process | 11 |
|  | reproduction | 2 |
|  | reproductive process | 2 |
|  | response to stimulus | 14 |
|  | signaling | 2 |
|  | single-organism process | 23 |
| cellular_component | cell | 32 |
|  | cell junction | 1 |
|  | cell part | 32 |
|  | extracellular region | 8 |
|  | macromolecular complex | 11 |
|  | membrane | 19 |
|  | membrane part | 7 |
|  | membrane-enclosed lumen | 1 |
|  | organelle | 29 |
|  | organelle part | 21 |
|  | symplast | 1 |
| molecular_function | binding | 22 |
|  | catalytic activity | 19 |
|  | electron carrier activity | 1 |
|  | enzyme regulator activity | 2 |
|  | nutrient reservoir activity | 1 |
|  | structural molecule activity | 2 |
|  | transporter activity | 3 |
