# Supplementary material for: Proteome Profiling of Paulownia Seedlings Infected with Phytoplasma
Source: Front Plant Sci. 2017 Mar 10;8:342. doi: 10.3389/fpls.2017.00342 (PMC5344924; doi:10.3389/fpls.2017.00342)
Supplement: Supplementary file 8 [file Table8.DOCX]

**Table S8 KEGG pathway analysis of the protein related to PaWB**

| # | KEGG Pathway | Count* | Pathway ID |
| --- | --- | --- | --- |
| 1 | Metabolic pathways | 13 | ko01100 |
| 2 | [Biosynthesis of secondary metabolites](file:///E:\cao\%E8%9B%8B%E7%99%BD%E8%B4%A8%E7%BB%84%E6%8A%A5%E5%91%8A\Report%20G\reportG_F12FTSCCKF0587_20140114\combined\Pathway\all_protein.htm#gene2) | 5 | ko01110 |
| 3 | [Carbon fixation in photosynthetic organisms](file:///E:\cao\%E8%9B%8B%E7%99%BD%E8%B4%A8%E7%BB%84%E6%8A%A5%E5%91%8A\report%20G%202015.8.12\Individuation_analysis\final\final_Pathway\final.htm#gene3) | 3 | ko00710 |
| 4 | [Photosynthesis](file:///E:\cao\%E8%9B%8B%E7%99%BD%E8%B4%A8%E7%BB%84%E6%8A%A5%E5%91%8A\report%20G%202015.8.12\Individuation_analysis\final\final_Pathway\final.htm#gene4) | 3 | ko00195 |
| 5 | Oxidative phosphorylation | 3 | ko00190 |
| 6 | Ribosome | 3 | ko03010 |
| 7 | Plant-pathogen interaction | 2 | ko00626 |
| 8 | Glyoxylate and dicarboxylate metabolism | 2 | ko00630 |
| 9 | Pyruvate metabolism | 2 | ko00620 |
| 10 | Nitrogen metabolism | 2 | ko00910 |
| 11 | Protein processing in endoplasmic reticulum | 1 | ko04141 |
| 12 | Fatty acid biosynthesis | 1 | ko00061 |
| 13 | Propanoate metabolism | 1 | ko00640 |
| 14 | Glycolysis / Gluconeogenesis | 1 | ko00010 |
| 15 | Starch and sucrose metabolism | 1 | ko00500 |
| 16 | Photosynthesis - antenna proteins | 1 | ko00196 |
| 17 | Other glycan degradation | 1 | ko00511 |
| 18 | Vitamin B6 metabolism | 1 | ko00750 |
| 19 | Phosphatidylinositol signaling system | 1 | ko04070 |
| 20 | Phagosome | 1 | ko04145 |
| 21 | Pentose and glucuronate interconversions | 1 | ko00040 |
| 22 | Porphyrin and chlorophyll metabolism | 1 | ko00860 |
| 23 | Proteasome | 1 | ko03050 |
| 24 | Ascorbate and aldarate metabolism | 1 | ko00053 |
| 25 | Glycerophospholipid metabolism | 1 | ko00564 |
| 26 | Phenylpropanoid biosynthesis | 1 | ko00940 |
| 27 | RNA degradation | 1 | ko00195 |

*: the number of the proteins related to PaWB involved in the corresponding pathway**.**
